# Beyond cognition: psychopathological sequelae of neonatal hypoxic-ischemic encephalopathy

**DOI:** 10.1186/s13052-025-02062-z

**Published:** 2025-07-15

**Authors:** Elisa Cainelli, Tiziana Battistin, Cristina Forest, Sara Puddu, Cristina Malaventura, Agnese Suppiej

**Affiliations:** 1 Department of General Psychology, University of Padova, Padova, Italy; 2 Department of Neuroscience and Rehabilitation, University of Ferrara, Ferrara, Italy; 3 Department of Medical Science, University of Ferrara, Ferrara, Italy

**Keywords:** Psychology, Internalizing symptoms, Somatic complaints, Perinatal asphyxia, Outcome

## Abstract

**Background:**

Since the introduction of therapeutic hypothermia (TH) for neonatal hypoxic-ischemic encephalopathy (HIE), mortality and severe morbidity have been reduced, but follow-up studies have shown a high incidence of neuropsychological impairments. By contrast, the possibility of psychopathological vulnerability in children with HIE has not been clearly addressed. Our study aims to investigate the presence of psychopathological symptoms by comparing in the prepuberal period a sample of children with a history of neonatal HIE to a control group.

**Methods:**

This is an observational cohort study with cross-sectional outcome assessment, where 76 HIE children (mean age 7.2 ± 1.84 years, range 5–12 years, 53.5% males) and 76 controls (mean age 7.8 ± 2.25 years, range 5–12 years, 51% males) were recruited. Exclusion criteria were the presence of major impairments. Parents completed the Child Behavior Checklist (CBCL) questionnaire. HIE group and controls have been compared, and the roles of age and sex have been evaluated.

**Results:**

A significantly higher percentage of psychopathological symptoms, in particular internalizing (*p* =.048) and somatic complaints (*p* <.001), in HIE children compared to peers have been found. The T scores of HIE and controls differed significantly for all CBCL subscales except for externalizing problems. An interaction between age and group in the variables strongly distinguishing HIE and controls has been found.

**Conclusion:**

Neurobiology of neonatal HIE may determine a vulnerability to developing psychopathological symptoms. Great attention should be paid to internalizing problems, given the risk of underestimating them in children and their potential to be precursors of most invalidating disorders at higher ages, such as depression.

**Supplementary Information:**

The online version contains supplementary material available at 10.1186/s13052-025-02062-z.

## Introduction

Neonatal hypoxic-ischemic encephalopathy (HIE) is one of the most important causes of death in the neonatal period, but also in survivors, many morbidities have been reported. Neonates suffering from HIE undergo therapeutic hypothermia (TH), which has proven to be effective in reducing mortality and severe morbidity [1–3] and has become the gold standard for the treatment of HIE since the end of the international trials in 2009 [3, 4].

The dramatic reduction in mortality allowed the researchers to shift their perspective from survival to quality of life and long-term outcomes. For this reason, great attention has been paid to the neurodevelopment of children with a history of neonatal HIE, highlighting how, despite the reduction in severe outcomes, these children can suffer from multiple isolated cognitive impairments [3–7].

Unfortunately, it will still take many years to fully understand the long-term consequences of using TH. The population of patients undergoing TH for neonatal HIE is, in fact, young: by becoming standard of care in 2009, the first neonates undergoing TH are currently teenagers and no data at present exist about the outcome at adult age. High-order cognitive functions and personality require many years to develop completely, and some reach their completion only in the third decade of life (in parallel with the development of prefrontal cortices) [8]. This concept takes on particular importance if we broaden the perspective to consider that three-quarters of mental disorders begin during adolescence and early adulthood [9] and that the number of children and adolescents suffering from psychological disorders in the contemporary era is rapidly increasing [10, 11].

Despite the shift in perspective in favor of a deeper view of disorders being transversal to every area of medicine, there is still much internal resistance. For example, neurologists are not ready to handle psychiatric issues. As confirmation, most studies on neonatal HIE focused on neurocognitive functioning but not on psychopathology. Physicians, patients, and families often overlook psychological and emotional symptoms, with disastrous consequences. Instead of considering psychological sequelae a result of neurological pathology or an abnormal social context, it would be more useful to investigate them as part of the process in action, guiding us to a more complex and less modular view of psychic functioning [12, 13]. The issue is important considering that functional abnormalities established during the perinatal period could manifest after a long time when it is more difficult to establish a pathogenic link [14]. Only recently, a systematic review evaluated the existing literature on psychological outcomes of perinatal HIE [15]. This review was not focused on TH and includes pre-TH studies and research comparing HIE with and without TH (but not controls). This work highlighted how perinatal HIE could be an important risk factor for a range of psychopathological challenges. Unfortunately, only a few studies investigate this aspect in children with a history of neonatal HIE treated with TH [16–18]. Alvarez-Garcia and colleagues, for example, in their exploratory study, found an increased risk of developing mood disturbances, in particular anxious, depressive, and aggressive symptoms [18]. In two previous research of our group, not focused on psychopathology, we found an abnormal autonomic nervous system functioning in HIE survivors, and that autonomic abnormalities were associated with socio-emotional skills [16, 17].

In the present study, we aimed to compare the psychopathological symptoms raised from the CBCL questionnaire in preadolescent children with a history of neonatal HIE treated with TH and control peers. Despite being a screening tool, quick and easy to administer, the CBCL has proven reliable [19]. In addition, it allows the differentiation of disorders into two major macro-areas: externalizing and internalizing disorders. Externalizing disorders concern those situations in which the child and adolescent’s distress spills outward, causing a disturbing situation in the surrounding environment. Belonging to this category, among others, are hyperactivity/attention disorders (ADHD) and conduct disorder. Unlike externalizing problems, which are overwhelmingly evident, internalizing problems are often misinterpreted or overlooked because the problems are developed and maintained internally (e.g., anxiety, withdrawal, and somatic complaints). They are marked by symptoms of overcontrol, that is, the child tends to regulate his or her emotional and cognitive states excessively and inappropriately.

Based on previous literature on HIE, we hypothesized that despite the treatment with TH and the reduction in severe impairments, perinatal adverse conditions may have resulted in increased psychopathological vulnerability. In particular, our interest was related to the presence of internalizing disorders, as externalizing ones, by their impactful nature, have always attracted the attention of scholars, and their prevalence and origin in children with a history of HIE have already been partly explored [15]. To investigate this issue, we look for the incidence of clinical conditions, as shown by overt pathological scores, as well as subclinical vulnerabilities, which may emerge from scores comparison between HIE and controls.

## Methods

### Participants

We prospectively recruited our participants in a cross-sectional observational cohort study between January 2018 and February 2024. Eligible participants were all children of at least 5 years who were born with HIE and undergone TH in our departments from February 2009 to July 2016. The families gave consent to be recalled for future studies on long-term outcomes. Exclusion criteria were: the presence of major impairments (neurosensory impairments, cerebral palsy, or epilepsy), diagnosis of congenital malformations after the neonatal period, inborn errors of metabolism, genetic syndromes, other medical comorbidities, certified intellectual disabilities, certified neurodevelopmental or psychiatric disorders, traumatic events or reported parental neglect, invalidating parental pathologies that emerged during clinical follow-up, and absence of parent consent. Among the initial database of 118 patients consecutively born and undergoing TH, 42 children were excluded because of exclusion criteria or dropout. We recruited a final convenience sample of 76 children. Children had a mean age of 7.2 years (SD 1.84, age range 5–12 years), and 40 (53.5%) were males. All the children fluently spoke Italian.

Inclusion criteria at birth for TH were (1) gestational age at birth ≥ 36 weeks; (2) any of the following: arterial umbilical cord or first blood gas analysis (within 1 postnatal hour) with pH 7.0, cord and base excess < 12, 10-min Apgar score < 5, or need for respiratory support at 10 min of life; and (3) moderate-to-severe encephalopathy within 6 h of birth. The newborn exclusion criteria were suspected or known congenital malformations and inborn errors of metabolism. TH was initiated as soon as possible after birth or at the time of referral from other hospitals and consisted of moderate whole-body hypothermia (target temperature 33–34 ^o^C) for 72 h followed by a rewarming rate of approximately 0.5 ^o^C/h. All patients received fentanyl infusion throughout TH to prevent discomfort and shivering (1–2 g/kg/h, with boluses as needed).

The controls were recruited in a primary school between March 2000 and January 2025. The project was presented, and families were asked to join freely. The exclusion criteria were the same as those for the HIE group. 76 healthy controls at a mean age of 7.8 years (SD 2.25, age range 5–12 years), and 39 (51%) males.

The ethical committee of our departments approved the study (Comitato Etico AOU Ferrara, University of Ferrara, prot. 636/2018/Disp/AOUF, approved on 01/23/2019; Comitato Etico della Ricerca Psicologica (Area 17), Università di Padova, prot. 4389, approved on 11/10/2021).

### Psychopathological evaluation

Parents completed the Italian adaption of the Child Behavior Checklist (CBCL) [19–21]. The CBCL is a multiaxial, empirically based set of measures that assess the child’s emotional, behavioral, and social problems over the previous six months. The CBCL questionnaire allows parents to report an array of symptoms using pencil-and-paper forms and provides information for a dimensional assessment of psychopathology. Questionnaires allow for the quick gathering of large amounts of information, and the CBCL is one of the most widely used questionnaires of child psychopathology [22, 23].

Considering our participants’ age range, we used two CBCL versions, the CBCL 1.5 -5 and the CBCL 6–18. The CBCL 1.5–5 is a parent-report used to analyze behavioral symptoms in preschoolers. It presents 100 items describing the presence and the frequency of specific behavior through a three-point Likert scale (0, not true; 1, sometimes true; 2, very true). The CBCL 1.5–5 consists of 3 summary scales (Internalizing, Externalizing, and Total Problems), 7 syndrome scales (Emotionally Reactive, Anxious/Depressed, Somatic Complaints, Withdrawn, Sleep Problems, Attention Problems, and Aggressive Behavior), 5 DSM-Oriented Scales, and 1 Stress Scale. The CBCL 6–18 is a 113-item parent report is used to assess child and adolescent psychopathology. Similarly, to the CBCL1.5–5, each item is rated on a three-point Likert scale. The items are categorized into the 3 summary scales (Internalizing, Externalizing, and Total Problems), 8 syndrome scales (anxious/depressed, withdrawn/depressed, somatic complaints, social problems, thought problems, attention problems, rule-breaking behavior, and aggressive behavior), 6 DSM-Oriented scales. Studies of the CBCL subscales indicated high retest reliability and adequate interrater reliability [19].

### Statistical analysis

For statistical analysis, we only considered summary and syndromic scales. The scores were converted into T-score (mean 50, standard deviation 10). In both CBCL versions (½–5 and 6–18) the T-score is considered clinically significant when 63 or above for summary scales and 70 or above for syndrome, while it is in the borderline clinical range when the value is between 60 and 63 for summary scales and between 65 and 70 for syndrome. Values under 60 for the summary scales or under 65 for other scales are considered not clinically significant. This categorization was performed to calculate the percentage of normal, borderline, and clinically relevant scores.

Since the preschool version (CBCL 1.5-5) was filled out by only a small proportion of subjects (19/76, 14%, for each group), the subscales not overlapping with those of the 6–18 version were not considered, and some subscales present only in the CBCL 6–18 version are missing for younger children (i.e., rule breaking, social problems, and thought problems). A sensitivity analysis was done by reanalyzing the data after removing the scores of the preschool version of the CBCL (i.e., data on younger participants).

To investigate pathological conditions as well as subclinical vulnerabilities, we compared groups and sex by using both the percentage of clinically impaired scores (Chi-square test) and the T-scores (Mann-Whitney U test). A Spearman rho was used to investigate the association between CBCL summary scales and age, and controlling for groups by performing a partial correlation.

Analyses have been corrected with false discovery rate correction for multiple comparisons. Finally, the combined predictive power of variables of interest (group and age) on meaningful CBCL scores has been performed using linear regression.

Statistics were determined using JASP (The JASP team 2018, version 0.19.2).

## Results

The T scores of HIE and controls differed significantly, after correction for false discovery rate (Benjamini-Hochberg Adjusted P value), for anxiety/depression (U = 2037.00, *p* =.009), withdrawal (U = 1766.55, *p* =.0004), somatic complaints (U = 1537.00, *p* =.0004), social problems (U = 1093.00, *p* =.010), though problems (U = 915.50, *p* =.0004), attention problems (U = 2061.00, *p* =.009), rule-breaking behavior (U = 1084.00, *p* =.009), aggressive behavior (U = 1963.50, *p* =.006), internalizing (U = 2130.00, *p* =.016), and total (U = 2027.00, *p* =.009) problems, but not for externalizing problems (U = 2318.00, *p* =.083). After the removal of the group of children younger than 6 years (19 in the HIE and 19 in the control group), all scales differed between groups, even externalizing symptoms (sensitivity analysis, see Supplementary Material). No significant differences have been found between male and female scores at the CBCL, also considering the groups separately.

By considering the percentage of total clinical (borderline + clinical elevations) scores on the CBCL questionnaire, HIE children and controls differed for somatic complaints and internalizing scales (Table 1), after discovery rate correction.

**Table 1:** Percentage of clinical scores on the CBCL questionnaire, Chi-square, and p-value after false discovery rate correction in HIE and control groups

CBCL	HIE Clinical %	Controls Clinical %	Chi-square
**Anxiety/depression**	17%	13%	χ^2^ = 1.16; *p* =.44
**Withdrawal**	26%	10%	χ^2^ = 5.75; *p* =.06
**Somatic complaints**	28%	7%	**χ**^2^ **= 11.16; *p* <.001**
**Social problems**	12%	12%	χ^2^ = 0.001; *p* =.97
**Though problems**	23%	12%	χ^2^ = 2.33; *p* =.33
**Attention problems**	17%	9%	χ^2^ = 1.81; *p* =.35
**Rule-breaking behavior**	7%	7%	χ^2^ < 0.001; *p* =.97
**Aggressive behavior**	9%	5%	χ^2^ = 3.73; *p* =.72
**Internalizing**	26%	10%	**χ**^2^ **= 5.75; *p* =.048**
**Externalizing**	10%	2%	χ^2^ = 2.74; *p* =.14
**Total**	12%	5%	χ^2^ = 1.89; *p* =.16

After controlling for group and after correction for false discovery rate (Benjamini-Hochberg Adjusted P value), the age resulted significantly correlated to internalizing (rho = 0.180, *p* =.042, Fisher’s z Effect Size = 0.182) and total problems (rho = 0.210, *p* =.033, Fisher’s z Effect Size = 0.213).

Finally, the predictive power of age, group, and interaction age * group on internalizing, somatic complaints, and withdrawal (significant before correction) has been performed using three linear regression models.

In the first linear model, only the interaction age*group (beta = 0.12, standard error = 0.04, *p* <.004) resulted in an independent predictor of somatic complaints. In the second linear model, the group (beta=-11.10, standard error = 3.99, *p* =.006) and the interaction age*group (beta = 0.17, standard error = 0.04, *p* <.001) resulted in independent predictors of withdrawal. In the third model, only the interaction age*group (beta = 0.14, standard error = 0.06, *p* =.023) resulted in an independent predictor of internalizing problems.

## Discussion

In the present study, we investigated whether children with a history of HIE treated with TH are at higher risk for psychopathology, by searching for clinically relevant conditions as well as subclinical vulnerabilities, which may emerge from comparison with healthy controls. By comparing the HIE group to control peers, we found significantly higher psychopathological symptoms, in particular, internalizing symptoms, somatic complaints, and withdrawal. The results also highlighted an interaction between belonging to the HIE group and age, which points out that in children with a history of HIE, psychopathological issues gradually manifest as they get older.

In our study, we used a screening tool, the CBCL. It is a quick and easy tool that could be consistently used in clinical settings to identify those individuals with a history of HIE who might benefit from more extensive psychopathological evaluation. The CBCL has been widely recognized as a reliable screening tool [22, 24], and therefore, it can reasonably be considered a valuable indicator of risk for psychopathology [24]. Perinatal conditions - among them perinatal asphyxia - are renowned risk factors for the development of neurodevelopmental disorders and psychopathology [14, 15, 25–28]. From the perspective of the biopsychosocial model of mental health, where life challenges and stress act on top of biological frailty [29, 30], we are led to hypothesize that perinatal health adversities, such as birth asphyxia, may allow a neurobiological vulnerability to later life adversities, with important implications for prevention. Despite this, the risk associated with neonatal HIE has been mainly evaluated in epidemiological studies and rarely investigated directly [15]. Considering the population treated with TH, even less information is present. Preliminary reports highlight a psychopathological and emotional vulnerability [16–18].

In the present study, externalizing symptoms do not appear to distinguish between groups as effectively as internalizing symptoms. This is surprising, considering that the area in which perinatal risk has been more extensively considered is behavioral disturbances, such as ADHD [26, 28]. The high presence of internalizing symptoms is information that must be managed very carefully. Most attention has always been paid to some neurodevelopmental disorders, like ADHD and autism spectrum disorder, given their strong impact on the social context of the child. Much less attention has historically been paid to internalizing disorders, which, because of their small impact on society, can be difficult to identify in children [31], and thus underestimated. Children with internalizing symptoms rarely exhibit significant behavioral difficulties at school, thus being rarely referred by their teachers for evaluation. Rather, they are often described as behaving well and eager to please [32], thus increasing the difficulties in identifying them. It is possible that the elapsing of time and the growing complexity of environmental demands bring to light problems that could remain hidden at younger ages.

Our sample is a group of selected children without disabilities. Thus, psychological disturbance could not be the consequence of limitations in the quality of life occurring in children with severe brain damage. We hypothesized that neonatal HIE, even in the absence of structural abnormalities and major neuromotor disabilities, could have determined an abnormal shaping of early neural circuitry and possibly also of the stress response [33, 34]. This dysfunctional pattern, however, may not become evident until the nervous system reaches a specific level of maturation and until demands arriving from the environment do not uncover latent vulnerabilities [35].

Indeed, complete maturation of the personality requires several years [36]. Psychopathological symptoms may be more subtle in younger children and manifest differently than at higher ages, for example, as inappropriate social attitudes or prominent physical manifestations [31, 33]. In our group, we found a high percentage of withdrawal, social problems, and somatic complaints, which refer to physical symptoms with no identifiable organic cause [37]. Somatic complaints have been suggested to reflect a limited ability to identify and express emotions [38], determining the use of maladaptive coping strategies and somatization. As a consequence, somatic complaints determine an increased risk of anxiety and depression symptoms across this developmental period [39] and in adulthood [40]. Withdrawal and social problems can then precipitate an already complex situation.

The neurobiological correlate of somatic complaints and other internalizing symptoms could reside in the abnormal functioning of the stress response system as a consequence of early overstimulation or dysregulation of the hypothalamic-pituitary-adrenal (HPA) axis and the autonomic nervous system (ANS) by HIE. TH and prolonged hospitalization in the neonatal intensive care unit could have a role in shaping an abnormal HPA and ANS functioning, given that both are programmed in utero and the early postnatal months [41, 42]. Therefore, we can speculate an interference from both HIE and associated conditions in developing and programming these systems. Similarly, at a more central level, there may be an imbalance of neurotransmitters and cortical and subcortical structures that regulate emotions [14].

This study has some limitations. The CBCL doesn’t represent an in-depth psychopathological evaluation, thus allowing only speculation about the real presence of psychopathology. Furthermore, given the age of some of the children, our CBCL is administered to parents, thus reflecting the numerous biases that the indirect evaluation can determine. An in-depth psychopathological assessment would have allowed us to better understand the meaning of these preliminary results. A complete and accurate history could have also brought out risk and protective factors, such as parenting styles. However, the CBCL could be the starting point for future studies; in clinical practice, it could be used as a screening and monitoring tool for infants and young children with a history of neonatal HIE.

Another limitation is the lack of control of confounding variables, such as parental style and socioeconomic status. These factors have a renowned influence on child development, and future studies must investigate their effects.

## Conclusions

In conclusion, the present study highlights that, compared to healthy peers, children with a history of neonatal HIE treated with TH are at higher psychopathological risk, also in the absence of neuromotor and sensory impairments. In particular, we found a high percentage of internalizing symptoms, which appear to increase with age. This counteracts the idea that as these children grow up, their problems diminish. It rather suggests the possibility that only after growth has occurred will we have a complete picture of the clinical features of these patients, whose biological frailty probably renders them particularly vulnerable to later life adversities.

## Electronic supplementary material

Below is the link to the electronic supplementary material.

Supplementary Material 1

Supplementary Material 2

## Data Availability

The datasets used and/or analysed during the current study are available from the corresponding author on reasonable request.

## Abbreviations

ADHD: Hyperactivity/attention disorders
ANS: Autonomic nervous system
CBCL: Child Behavior Checklist
HIE: Hypoxic-ischemic encephalopathy
HPA: Hypothalamic-pituitary-adrenal
TH: Therapeutic hypothermia
