# Supplementary material for: Beyond cognition: psychopathological sequelae of neonatal hypoxic-ischemic encephalopathy
Source: Ital J Pediatr. 2025 Jul 15;51:228. doi: 10.1186/s13052-025-02062-z (PMC12261663; doi:10.1186/s13052-025-02062-z)

P-values before and after False Discovery rate of Mann-Whitney U-tests comparison between HIE and controls without children younger than 6 years.


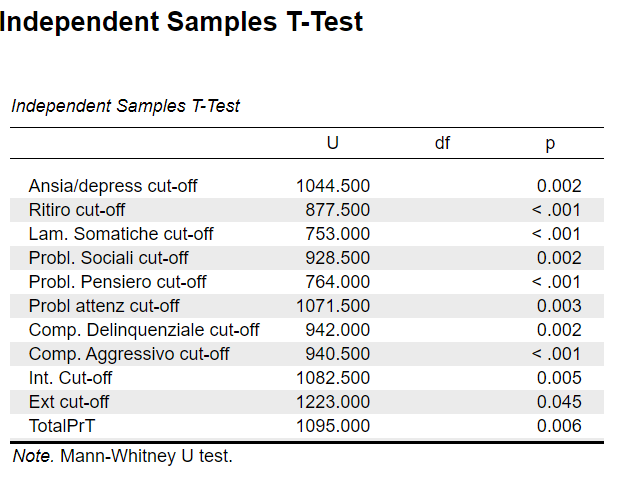


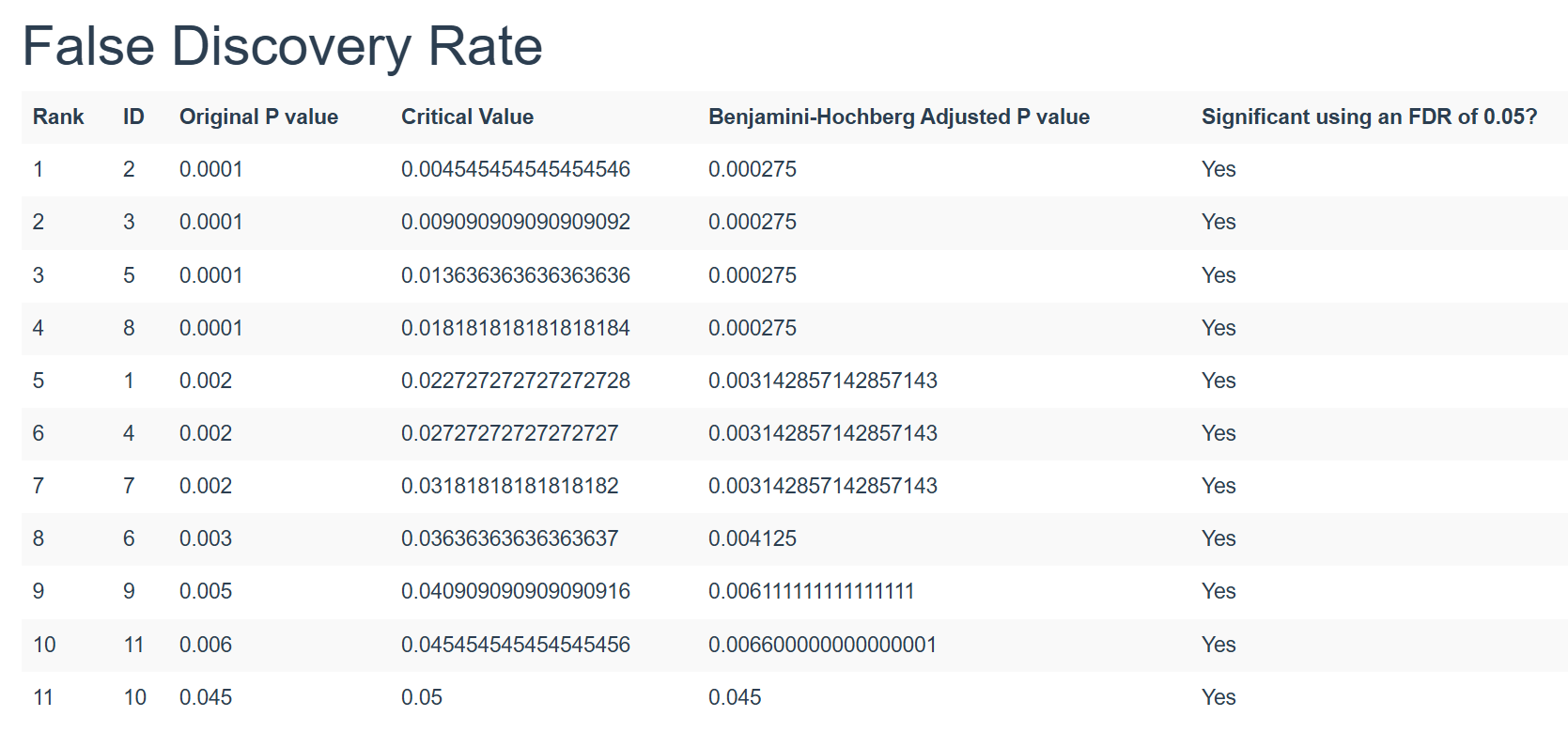

Supplement: Supplementary file 1 — Supplementary Material 1 [file 13052_2025_2062_MOESM1_ESM.docx]
