# Supplementary material for: Beyond cognition: psychopathological sequelae of neonatal hypoxic-ischemic encephalopathy
Source: Ital J Pediatr. 2025 Jul 15;51:228. doi: 10.1186/s13052-025-02062-z (PMC12261663; doi:10.1186/s13052-025-02062-z)
Supplement: Supplementary file 2 — Supplementary Material 2 [file 13052_2025_2062_MOESM2_ESM.pdf]

24.04.2025

Dear Editor,

We ask you to consider our revision of the article "Beyond Cognition: Psychopathological Sequelae of Neonatal Hypoxic-Ischemic Encephalopathy”, written by Dr. Elisa Cainelli, Prof. Tiziana Battistin, Dr. Cristina Forest, Dr. Sara Puddu, and Prof. Agnese Suppiej.

We considered all issues mentioned in the reviewers' comments carefully; every change made in response to their comments in the manuscript have been explained in the comments.

Please address all correspondence concerning this at cainelli.elisa@gmail.com or agnese.suppiej@unife.it.

Thank you for consideration of this manuscript for publication in the Italian Journal of Pediatrics.

Best Regards,

Dr. Elisa Cainelli,
corresponding author https://orcid.org/0000-0002-5039-4680
Department of General Psychology, University of Padova,
Via Venezia, 8 – 35133, Padova, Italy.
Email: cainelli.elisa@gmail.com
